# Biochemical characterization of lipase from *Cryptococcus albidus* (D24) and its application in sugar fatty acid ester synthesis

**DOI:** 10.1007/s00203-025-04369-1

**Published:** 2025-06-13

**Authors:** Duygu Elif Yilmaz, Ceyda Kula, Hasan Demirci

**Affiliations:** 1 Department of Bioengineering, Marmara University, Istanbul, Turkey; 2 Department of Nephrology and Medical Intensive Care, Charité— Universitätsmedizin Berlin, Berlin, Germany; 3 Department of Clinical Infection, Microbiology and Immunology, University of Liverpool, Liverpool, UK; 4 Department of Functional Anatomy, Charité—Universitätsmedizin Berlin, Berlin, Germany; 5 Department of Medical Biology, Faculty of Medicine, Ankara Yildirim Beyazit University, Ankara, Turkey

**Keywords:** Lipase, Esterification, C*ryptococcus albidus*, Sugar fatty acid ester

## Abstract

Microbial lipases are widely used in industrial applications. The lipase enzyme from *Cryptococcus albidus* (D24) strain was biochemically characterized and evaluated for its potential in catalyzing esterification reactions, particularly in the synthesis of sugar fatty acid esters. The enzyme demonstrated enhanced activity in solvents like acetone, isopropanol, and dimethylformamide. The effects of different metal ions on the stability of the enzyme were also evaluated, and the results revealed an increased activity with Mn²⁺, K^+^ and Co^2+^. The molecular weight of the D24 lipase was determined to be 36.31 kDa, placing it within the range of other yeast-originated lipases. The kinetic parameters, including Km and Vmax, were calculated to be 1.58 × 10^−4^ mM *p*-nitrophenyl palmitate (*p*NPP) and 26.31 U/min respectively, according to the Lineweaver–Burk plot. The enzyme exhibited promising results in catalyzing the esterification reaction to yield L-proline-glucose ester, as well as the synthesis of fructose monopalmitate. Thin layer chromatography (TLC) analysis of the lipase-catalyzed synthesis of fructose monopalmitate showed that, from 24 to 40 h, fructose monopalmitate concentration increased from 4.5 to 8.4% (w/w of the reaction mixture). These findings suggest the potential of D24 lipase for applications in synthesis of industrial products. This study is the first to report on the biochemical properties of the D24 lipase, offering a novel and sustainable enzymatic alternative for the future biotechnological applications.

## Introduction

Lipases (EC 3.1.1.3) are triacylglycerol hydrolases that catalyze a wide range of reactions such as esterification, interesterification, transesterification, and hydrolysis (Melani et al. 2020). Although lipases are ubiquitously found in various organisms, from bacteria to higher organisms like animals, microbial lipases hold greater potential due to their high production yields, convenient cultivation processes, organic solvent stability, chemoselectivity, regioselectivity, and enantioselectivity (Bharathi and Rajalakshmi 2019; Singh and Jana 2023).

Among microbial lipases, fungal lipases have gained significant interest since the 1950s due to their superior biochemical properties, including high thermal and pH stability, broad substrate specificity, and compatibility with organic solvents, which enhance their utility in downstream processing applications (Gupta et al. 2015; Mahfoudhi et al. 2022; Abdelaziz et al. 2025). Their extracellular nature facilitates cost-effective recovery and purification, especially in batch fermentation systems, enhancing their feasibility for large-scale use. Compared to bacterial lipases, fungal lipases are generally more stable and versatile, providing a broader operational window for industrial processes including chemical, pharmaceutical, and biodiesel production (Kumar et al. 2023). Among these processes, the enzymatic synthesis of sugar fatty acid esters (SFAEs) has emerged as a promising application. SFAEs are non-ionic surfactants with wide-ranging applications in the food, cosmetic, and pharmaceutical industries due to their emulsifying, antimicrobial, and biodegradable properties (Li et al. 2024). Enzymatic synthesis of SFAEs, particularly using fungal lipases, is favored over chemical synthesis because it operates under milder conditions, yields fewer byproducts, and ensures greater specificity (Zheng et al. 2015; Pérez et al. 2017; Tracy et al. 2023).

As a result, in recent years, the demand for lipases to be used in various industries has been increasing which leads scientists to investigate novel resources. However, due to the wide spectrum of lipases, it is necessary to determine the unique properties of each enzyme to understand their biotechnological significance. For instance, solvent stability, thermostability, or pH stability provide critical advantages during industrial production stages (Yilmaz et al. 2014; Yilmaz and Sayar 2015).

Lipase-producing yeasts can be naturally found in oil-contaminated soil as a result of production waste. Previously, Yalçin et al. (2014) isolated lipase-producing yeast strains from petroleum-contaminated sludge. The yeast strains were identified via molecular characterization experiments as *Candida parapsilosis* (D3), *Rhodotorula mucilagnosa* (D17), *Cryptococcus albidus* (D24), and *Cryptococcus albidus* (D27). Among these strains, the D24 lipase from *C. albidus*, showed significantly high lipase activity. Therefore, in the present work, D24 lipase was further characterized in order to discover its potential in industrial use. Subsequently, esterification reactions were conducted to synthesize various sugar esters using the novel D24 lipase from *C. albidus.*

## Materials and methods

### Yeast strains, cultivation and lipase production

In a previous study, the yeast strains *Candida parapsilosis* (D3), *Rhodotorula mucilaginosa* (D17), *Cryptococcus albidus* (D24), and *Cryptococcus albidus* (D27) were isolated from petroleum-contaminated sludge and their molecular characterization was performed using RFLP of ITS1-5.8 S-ITS2 and 18 S rRNA along with sequence analysis of D1/D2 domain of 26 S rRNA (Yalçin et al. 2014). Subsesquently, these species were kindly provided by Ege University, Faculty of Science, Department of Biology, Basic and Industrial Microbiology Section for further research on their lipase production profile, biochemical characterization, and potential industrial applications.

Yeast strains were initially inoculated into a precultivation medium containing 0.3% (w/v) malt extract (Merck), 0.3% (w/v) yeast extract (Merck), 0.5% (w/v) peptone (Merck), and 1% (w/v) glucose (Biomérieux). The cultures were incubated overnight (16 h) with shaking (180 rpm) at 28 °C. Subsequently, 2% of the suspension (v/v) was inoculated into a lipase production medium composed of 0.1% yeast extract (w/v; Merck), 3% peptone (w/v; Merck), 0.05% MgSO_4_.7H_2_O (w/v, Merck), 0.1% KH_2_PO_4_ (w/v; Sigma-Aldrich), 0.3% NaNO_3_ (w/v; Merck), 2% tributyrin (v/v; Sigma-Aldrich), with the pH was adjusted to 7.0. The inoculated suspension was incubated at 28 °C with shaking at 180 rpm. Growth was monitored spectrophotometrically at 600 nm (OD₆₀₀). At specific time intervals, extracellular crude lipase was separated from yeast cells by centrifuging at 10,000 rpm for 20 min followed by lipase activity assay as described previously (Rapp and Backhaus 1992), using *p*-nitrophenyl palmitate (*p*NPP) as the substrate. All enzyme assays were performed in triplicate. Protein concentrations were determined using the Bradford method, with bovine serum albumin as the standard (Bradford 1976).

### Partial purification of lipase

To increase the concentration, extracellular lipase was partially purified. Consequently, lipase solution was partially cleared of residual medium salts and small proteins lighter than 10 kDa. For partial purification, crude extracellular lipase was dialysed at 4 °C in 50 mM sodium acetate (OmniLife Scince) buffer (pH: 5.6) for 16 h. Dialysed lipase was concentrated by filtering it with centrifugation at 10,000 rpm in the 10 kDa-cut-off tubes (Merck, Amicon).

### Determination of molecular weight

Denatured proteins were separated via sodium dodecylsulphate polyacrylamide gel electrophoresis (SDS-PAGE) using a 5% (w/v) stacking gel and a 12% (w/v) separating gel. A total of 60 µg of the partially purified enzyme was loaded into each well. Electrophoresis was performed at a constant voltage at 110 V for 90 min in tris-glycine buffer, pH: 8.3. After the electrophoresis, one of the two separating gels was used to visualize the protein bands via Coomassie Brillant Blue G-250 (Amresco) staining. The other separating gel was incubated overnight in renaturation buffer as described by Sifour et al. (2010). The molecular weight of the enzyme was determined using the Thermo Scientific Protein Molecular Weight Marker 26610, which includes seven proteins ranging from 14.4 to 116 kDa. Next, relevant band areas were cut out of the gel and incubated in 1 mL substrate solution for 30 min at 37 °C, the reaction was stopped by the addition of stop solution and followed by the measurement of the lipase activity.

### Determination of kinetic parameters

Lipase activity was measured in different substrate concentrations of *p*NPP from 5 × 10^−5^ mg/mL (1.3 × 10^−4^ mM) to 45 × 10^−5^ mg/mL (1.2 × 10^−3^ mM). Enzyme activity was detected at 37 °C and pH: 5.6. The Km and Vmax values of the enzyme for *p*NPP substrate were calculated according to Michaelis-Menten kinetics using a Lineweaver-Burk plot.

### Synthesis procedures

#### Synthesis of L-prolyl-D-glucose sugar esters

Enzymatic esterification reactions were carried out according to the method described by Lohith (2006). Briefly, esterification reaction of L-proline and D-glucose catalyzed by lyophilized D24 lipase was carried out in blue cap bottles by reacting 0.001–0.008 mol of L-proline and 0.001–0.008 mol of D-glucose along with 100 mL of CH_2_Cl_2_:DMF (v/v, 90:10, 40 °C) in presence of 0.018–0.25 g of lipase (10–50% by weight of glucose) at 220 rpm shaking for a period of 72 h (Lohith 2006). The addition of the lipase initiated the reaction. Reaction mixtures were also examined with no lipase as negative control, and they were handled in the same manner. During this period, the reaction conversion was controlled by thin layer chromatography (TLC) daily. TLC analysis was performed on a 20 × 20 cm silica gel plate (mesh size 60 − 120) using isopropanol: water (4:1) as the developing system. The plates were sprayed with ninhydrin to develop amino acid spots. The reaction mixture was analyzed by high performance liquid chromatography (HPLC, Agilent Technologies) using a C18 column, acetonitrile and water in the ratio of 20:80 (v/v) and detection was at 210 nm using UV detector (Somashekar and Divakar 2007).

#### Synthesis of fructose fatty acid ester

Fructose fatty acid ester synthesis was carried out according to the method described by Kumar (2012). Briefly, the reaction mixture consisted of 0.6 mmol fructose as sugar, 0.6 mmol palmitic acid as fatty acid, 2 g of lyophilised D24 lipase with 5 mL of acetone as solvent. The reaction mixture was shaken at 125 rpm at 30 °C (Kumar 2012). The addition of the lipase initiated the reaction. Reaction mixtures were also examined with no lipase as negative control, and it was handled in the same manner. Samples were analyzed qualitatively by TLC. TLC analysis was performed on silica gel plates 60 F254 using chloroform: methanol: acetic acid: water (70:20:8:2) as the developing system. The plates were sprayed with 50% sulphuric acid and heated at 110 °C for 5 min to develop sugar and sugar ester spots (Cao et al. 1996).

## Results and discussion

In this study, a novel extracellular lipase from *Cryptococcus albidus* was characterized and assessed for its potential in biotechnological applications. The findings cover a broad range of experimental investigations, including enzyme production, activity screening, partial purification, and biochemical characterization. Additionally, the thermostability and pH stability of the enzyme were evaluated, followed by its application in the synthesis of sugar fatty acid esters. Each result is discussed in light of previous studies, providing a comprehensive understanding of the D24 lipase’s industrial relevance and performance.

### Production of lipase enzyme from *Cryptococcus albidus* D24

**Fig. 1:**
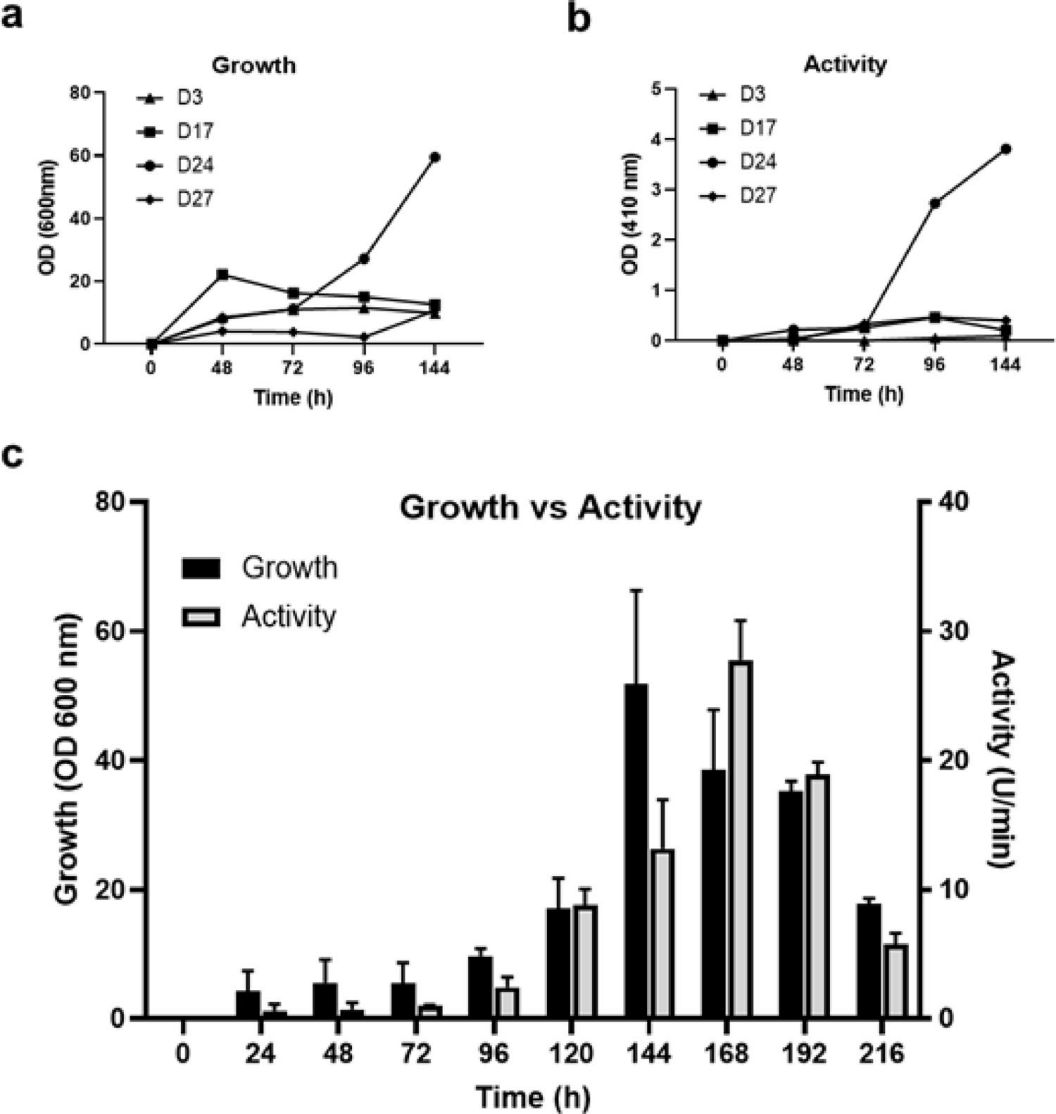
Growth and lipase activity profiles of *Candida parapsilosis* (D3), *Rhodotorula mucilaginosa* (D17), *Cryptococcus albidus* (D24), and *Cryptococcus albidus* (D27) for 144 h (**a**, **b**). Growth and lipase activity profiles of the *Cryptococcus albidus* (D24) for 216 h (**c**). Data are presented as mean ± standard deviation

The growth and the lipase activity profiles of the D3, D17, D24 and D27 were screened briefly (Fig. 1a, b). The screening results revealed exceptionally high D24 lipase activity compared to the other strains; therefore, the activity and growth profile of D24 lipase were further characterized. Figure 1c shows that the highest growth level was detected in 144th hour, whereas the peak point of the lipase activity was in 168th hour. Based on the results, the crude lipase was harvested at the 168th hour for further experiments. A similar lipase activity profile was also screened by Tiwari et al. (2011) in *C. albidus* and comparably their group found the highest lipase activity in 120th hour.

Next, a partial purification methodology was performed to increase the specific activity of the D24 lipase. The crude D24 lipase was dialysed in sodium acetate buffer (pH: 5.6) overnight at 4 °C followed by a concentration step with filtering centrifuge tubes. The results showed that partial purification step increased the specific activity almost 4 fold (Table 1). Further characterizations were performed using dialized and concentrated D24 lipase.

**Table 1:** Effect of partial purification on specific activity of D24 lipase

	Total protein, mg	Total activity, U	Specific activity, U/mg	Fold
Crude D24	33.46	528.35	15.79	1.00
Dialysed and concentrated D24	6.82	420.12	61.63	3.90

### Effect of temperature and pH on D24 lipase

**Fig. 2:**
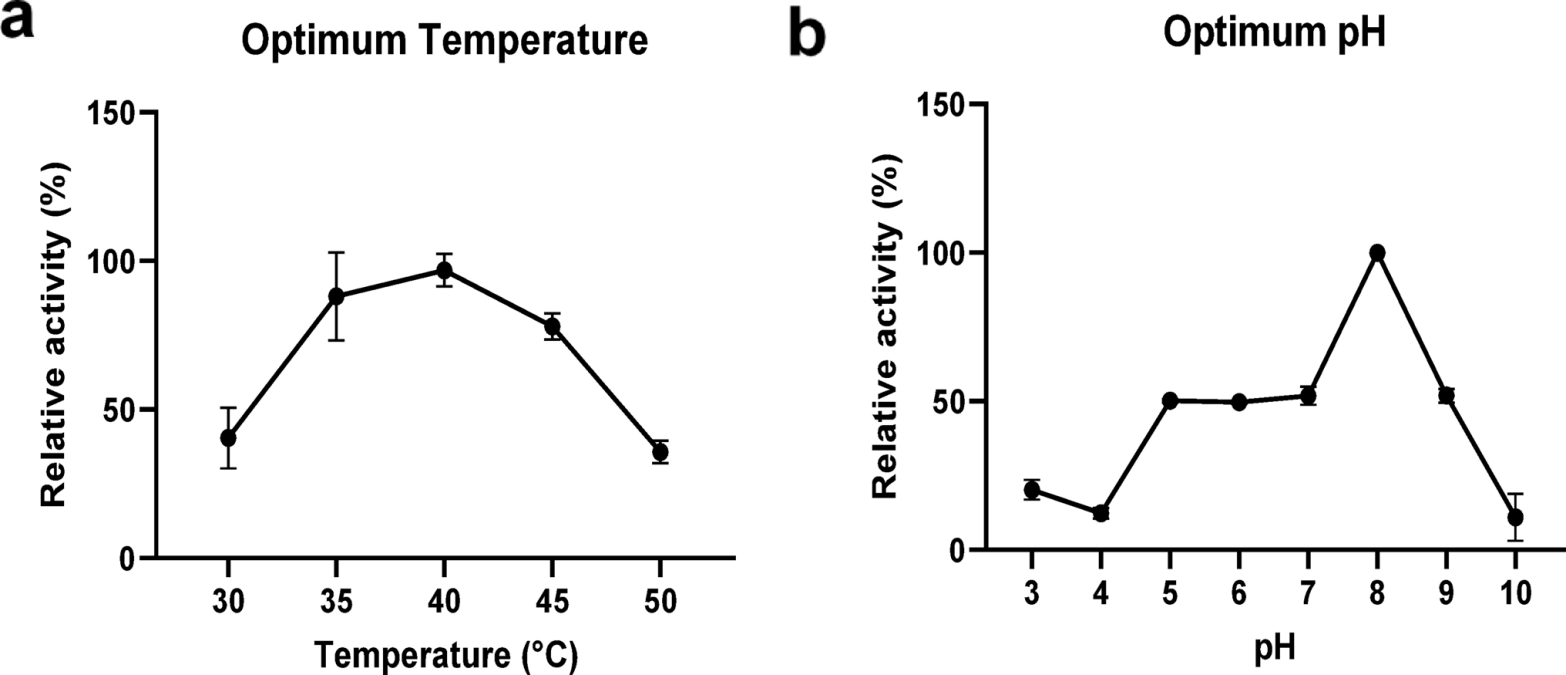
Optimum temperature (**a**) and optimum pH (**b**) profiles of the *Cryptococcus albidus* (D24) lipase. Data are presented as mean ± standard deviation

The D24 lipase exhibited activity between 30 °C and 50 °C, with an optimal temperature of 40 °C (Fig. 2a). Its optimal pH was determined to be 8, within a tested range of 3 to 10 (Fig. 2b). Lipases from various yeast species display a broad spectrum of optimal temperatures and pH values (Kiran et al. 2016). For instance, lipases from *Hyphopichia wangnamkhiaoensis* and *Yarrowia deformans* exhibit optimal activity at pH 8.0, with significant activity between pH 7.5 and 9.0 (Romo-Silva et al. 2024). Conversely, the lipase from *Candida albicans* demonstrates optimal activity at a lower temperature of 25 °C and pH 8.0 (Lan et al. 2011). Given this variability, the D24 lipase can be classified as a mesophilic alkaline lipase, aligning with enzymes that function optimally in moderate temperature ranges and alkaline pH conditions.

**Fig. 3:**
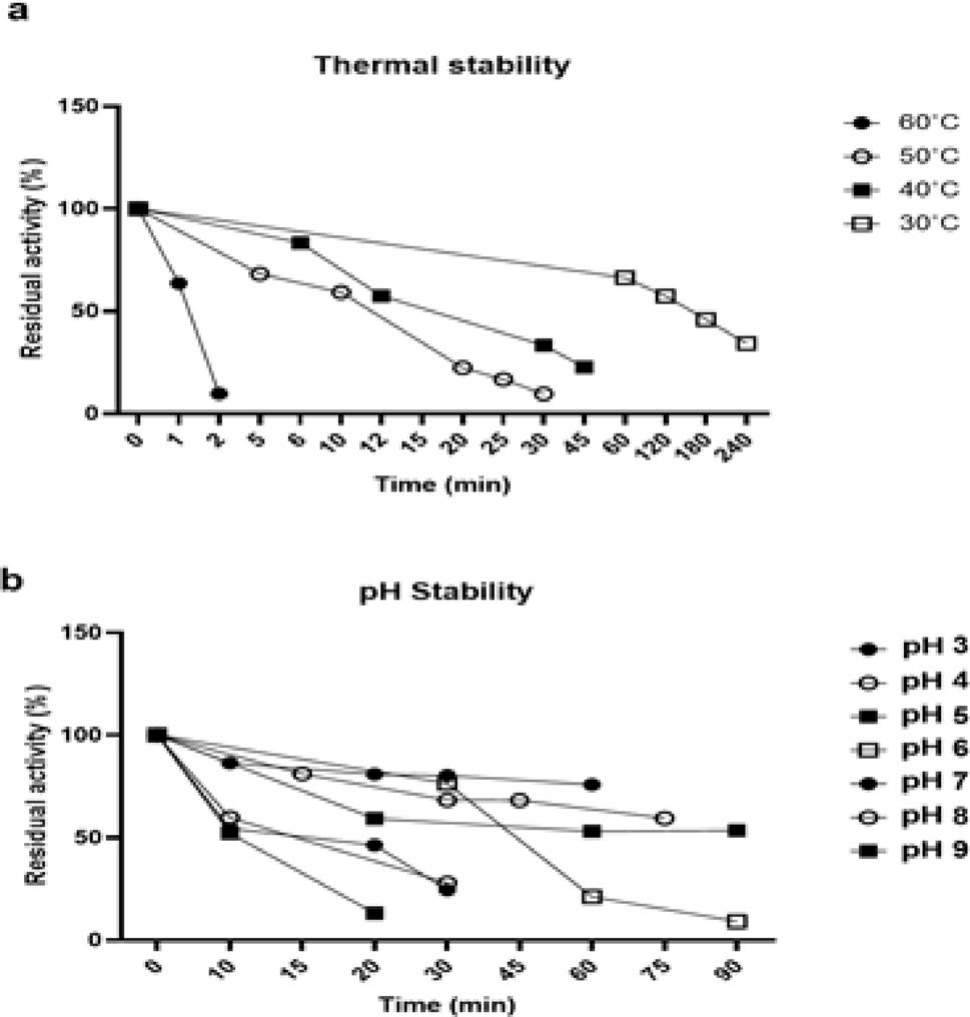
Thermal stability (**a**) and pH stability (**b**) profiles of the *Cryptococcus albidus* (D24) lipase, evaluated over a temperature range of 30–60 °C and pH range of 3–9, respectively. Data are presented as mean ± standard deviation

Next, the thermostability and pH stability of the D24 lipase were evaluated. Time profiles of D24 lipase stability at different temperatures are shown in Fig. 3a. The enzyme retained 34% of its initial activity at 30 °C after 240 min of incubation. The half-life time at this temperature was calculated to be almost 3 h. However, above 30 °C, activity of D24 lipase sharply decreased. The half-life of D24 lipase at 40, 50 and 60 °C were calculated to be 27, 9 and 0.59 min, respectively. Comparing the D24 lipase with lipase from *C. albidus* (Tiwari et al. 2011), D24 lipase was not found to be a thermostable lipase. Nevertheless, thermostability could be enhanced via immobilization of the lipase (Dosanjh and Kaur 2002).

The enzyme showed the highest stability at pH 3.0 and 5.0 values and the activity significantly dropped at pH above 5.0 (Fig. 3b). Enzyme lost 20%, 30% and 40% of its activity at pH 3.0, 4.0 and 5.0 within the first 30 min. Afterward, no activity loss was observed with prolonged incubation. Although fungal lipases generally work optimally in natural pH values (Sharma et al. 2016), D24 lipase was found to be more stable in a range of 3–5. Therefore, D24 lipase could be considered as acid-tolerant lipase.

The optimal pH for D24 lipase activity is pH 8.0, while the enzyme is most stable between pH 3.0 and 5.0. Although this may initially appear contradictory, this phenomenon is common and well-documented in enzyme biochemistry. It is important to note that the optimal pH for catalytic activity and the pH for maximal structural stability are not necessarily the same, as they are influenced by different molecular determinants. Catalytic activity depends on the precise arrangement and protonation states of amino acids at the active site, while structural stability relates to the overall folding and resilience of the enzyme’s tertiary structure (Talley and Alexov 2010).

### Effect of organic solvents on lipase activity

Organic solvents are widely used in lipase-catalyzed production processes. The use of organic solvents in lipase-catalzed reactions has many advantages for instance, increasing the activity, stability, substrate solubility and shifting the reaction equlibrium towards to the sythetesis of the product. Thus, it is very important to investigate the stability of the enzyme in the presence of organic solvents (Sharma and Kanwar 2014). Therefore, the effect of acetone, chloroform, isoprophanol, ethanol, glycerol, dimethylformamide (DMF), dimethyl sulfoxide (DMSO), methanol, *tert*-butyl alcohol and acetonitrile on D24 lipase activity was investigated. The stability of D24 lipase in the presence of these organic solvents was investigated by measuring residual activity of the enzyme in the presence of 5% or 10% organic solvent, relative to the control without any solvent (100%) after 30 min of incubation.

For all the organic solvents tested in the present work, the activity increased with the increase of organic solvent concentration except for acetonitrile after 30 min incubation period (Table 2). Increasing the acetonitrile concentration from 5 to 10% caused a decrease in residual D24 activity from 86.97 to 63.82%, respectively. On the other hand, the highest degree of activation was obtained in acetone, isopropanol, glycerol and DMF at 10% of concentration showing a relative activity of 110.57, 104.59, 101.27 and 100.38, respectively. Increasing concentrations of ethanol did not change the D24 lipase activity, whereas the activity considerably increased with an increase in DMSO, methanol and *tert*-butyl alcohol.

These findings align with studies on yeast lipases. For instance, the lipase from *Candida viswanathii* demonstrated increased activity in the presence of glycerol (135.4%), DMSO (111.7%), propylene glycol (111.5%), ethanol (106.2%), *n*-hexane (105.9%), and methanol (105.1%). However, it exhibited reduced activity with acetonitrile (86.2%), acetone (90.5%), and isopropanol (78.3%) (de Almeida et al. 2016). Similarly, the lipase from *Cryptococcus diffluens* D44 showed enhanced activity in 20% acetonitrile, achieving a residual activity of 173.24% (Yilmaz and Sayar 2015). These parallels suggest that D24 lipase shares solvent tolerance characteristics with other yeast-derived lipases, making it a promising candidate for industrial applications requiring robust enzyme performance in various solvent systems.​.

Previous studies have shown that low to moderate concentrations of methanol can enhance substrate solubility and serve as an acyl acceptor in biodiesel production reactions (Najjar et al. 2021). Eventhough organic solvents like methanol are generally known to exert an inhibitory effect on lipases, there are naturally occurring microbial lipases—particularly from genera such as *Pseudomonas* and *Burkholderia*—that exhibit notable tolerance to organic solvents (Sharma and Kanwar 2014; Lotti et al. 2015). Santambrogio et al. (2013) demonstrated that while lower concentrations of methanol led to high conversion yields, higher concentrations (e.g., 87%) resulted in strong inhibitory effects due to protein aggregation.

Organic solvent stable lipases are very crucial for the manufacturing of industrial products such as SFAEs since their reaction media usually require organic solvents and very low amount of water (Gumel et al. 2011). Due to its organic solvent stability, D24 lipase has a good potential for industrial benefit. Organic solvent stability allows these enzymes to perform efficiently and effectively under conditions that would typically denature or inactivate other enzymes (Ogino and Ishikawa 2001). D24 lipase stands out due to its high stability in the presence of organic solvents, making it a promising candidate for industrial applications. The robust nature of D24 lipase ensures that it can withstand harsh industrial conditions, thereby enhancing the efficiency and cost-effectiveness of the manufacturing processes.

**Table 2:** The effect of organic solvents on D24 lipase stability

Organic Solvent	Concentration, %	Relative Activity, %
Control	-	100
Acetone	5	84.27
	10	110.57
Chloroform	5	55.87
	10	62.55
Isoprophanol	5	84.86
	10	104.59
Ethanol	5	78.05
	10	79.24
Glycerol	5	91.20
	10	101.27
Dimethylformamide	5	83.10
	10	100.38
Dimethyl sulfoxide	5	70.89
	10	92.61
Methanol	5	62.91
	10	94.52
*tert*-Butyl Alcohol	5	63.03
	10	92.99
Acetonitrile	5	86.97
	10	63.82

### Effect of metal ions on lipase activity

The effect of metal ions on D24 lipase stability was investigated in two different concentrations (50–100 µg/mL) with an incubation period of 1 h. The results are given in Table 3. The presence of metal ions at a concentration of 50 µg/mL did not cause a major change in the activity of D24 lipase. However, 100 µg/mL concentration of Ca^2+^, Mg^2+^, Mn^2+^, K^+^, Fe^3+^, Co^2+^ and NH_4_^+^ increased the lipase activity after 1 h of incubation time. In particular, 100 µg/µL of Mn^2+^ increased the activity of D24 lipase up to 161.79%. However, the presence of sodium ions at 100 µg/mL concentration caused D24 lipase activity to drop drastically from 99.20 to 34.15%. Increasing Zn^2+^ concentration to 100 µg/mL caused a decrease in D24 lipase activity slightly. It can be suggested that higher sodium, zinc and silver ion concentrations could alter the D24 lipase conformation (Liu et al. 2008).

No general prevailing behaviour has been reported for metal ion effect on lipases, as different metal ions alter the enzyme conformation distinctly. Eventhough lipases generally do not require metal ions as cofactors, metal ions can stimulate or inhibit the activity of lipases (Lu et al. 2013). This fact highlight the versatility of the properties of lipases from different sources and recapitulate the need for individual characterisation of enzymes from new sources. The versatile properties of D24 lipase, such as organic solvent stability, stability in the presence of metal ions make *Cryptococcus albidus* D24 lipase suitable for different industrial applications (Yilmaz 2016).

**Table 3:** The effect of different metal ions on D24 lipase activity

Metal ions	Relative activity, %	
	50 µg/mL	100 µg/mL
Control	100.00	100.00
Ca^2+^	92.20	119.51
Ni^+^	92.60	102.44
Mg^2+^	90.40	116.26
Mn^2+^	104.00	161.79
Na^+^	99.20	34.15
K^+^	93.00	138.21
Cu^2+^	94.60	98.37
Zn^2+^	99.80	78.86
Fe^3+^	92.60	115.45
Co^2+^	92.60	138.21
NH_4_^+^	93.40	119.51
Ag^+^	101.60	91.06

### Determination of molecular weight and kinetic parameters of D24 lipase

**Fig. 4:**
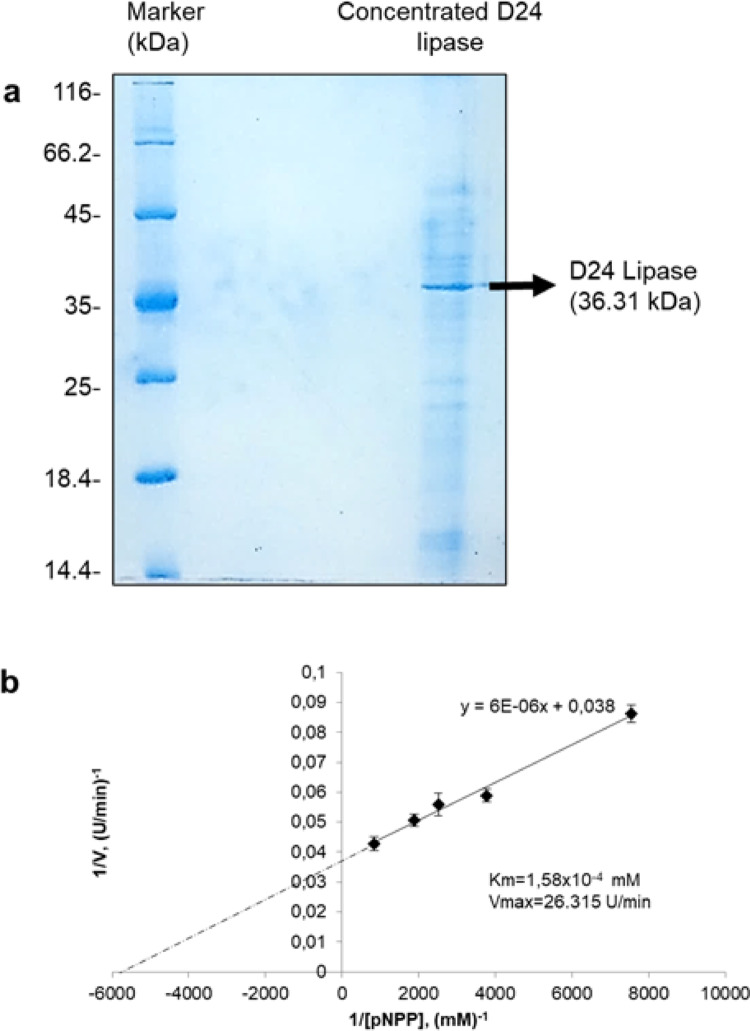
Evaluation of the molecular weight of the *Cryptococcus albidus* (D24) lipase by SDS PAGE (**a**). Lineweaver–Burk Plot of the *Cryptococcus albidus* (D24) lipase (**b**)

Renaturated gels were used to determine the molecular weight of the dialysed and concentrated D24 lipase. In order to determine which band belongs to lipase, all protein bands were excised and subjected to lipase activity assay. The results showed that the prominent band (indicated with an arrow) corresponds to D24 lipase. The correspondent band has been measured to have a molecular weight of 36.31 kDa (Fig. 4a).

Multiple form of microbial lipases have been repoted in the literature due to post–transcriptional processing, deglycosilation or existence of different genes. Most of the yeast originated lipases have molecular weight in the range of 33–65 kDa (Vakhlu and Kour 2006). The molecular weight of the D24 lipase, as 36.31 kDa, places it within the previously reported range.

Generally, lipases obey the Michaelis–Menten kinetics (Sharma et al. 2001). Therefore Km and Vmax values of D24 lipase was determined from the Lineweaver–Burk plot and calculated as 1.58 × 10^−4^ mM *p*NPP for Km and 26.31 U/min Vmax (Fig. 4b). Lower a Km represents higher affinity of the enzymes; the Km values of the most industrially relevant lipase enzymes range between 10^−1^ and 10^−5^ M (Sharma et al. 2001). D24 lipase can be regarded as industrially relevant considering the Km values.

### Esterification reactions

**Fig. 5:**
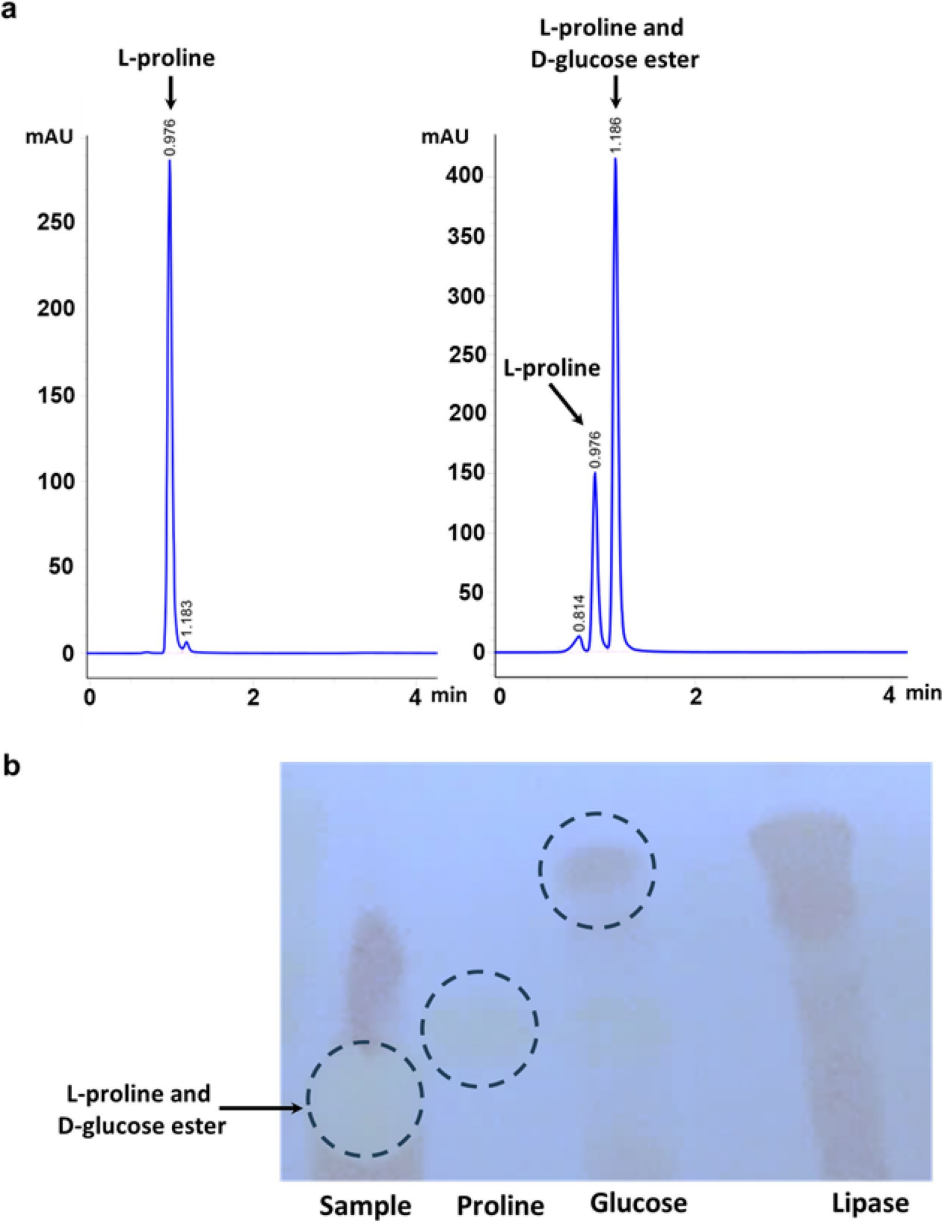
HPLC chromatogram of L-proline, reaction mixture of L-proline and D-glucose esterification reaction catalyzed by *Cryptococcus albidus* (D24) lipase (**a**). TLC of L-proline-D-glucose ester (**b**). Proline, glucose and lipase standards used as controls

Sugar ester synthesis of L-proline with D-glucose was carried out using the D24 lipase enzyme under optimal conditions. The reaction was conducted in blue cap bottles by reacting same amount of L-proline and D-glucose along with 100 mL of CH_2_Cl_2_:DMF (v/v, 90:10, 40 ^◦^C) in the presence of lipase (10–50% by weight of glucose) at 220 rpm for a period of 72 h. During this period, the conversion of the reaction was daily controlled by TLC and was measured by HPLC. Chromatographic analysis of L-proline-glucose ester was carried out to evaluate the yield of the esterification reaction. HPLC chromatogram (Fig. 5a) and TLC (Fig. 5b) images are shown in Fig. 5 (a-b).

Another esterification reaction was conducted for the synthesis of fructose fatty acid esters. The reaction methodology was adapted from Šabeder et al. (2006). Palmitic acid and fructose were used as substrates to perform the lipase-catalyzed esterification reaction. The reaction was performed in a solvent system consisting of acetone for 40 h, at 30 °C with shaking at 125 rpm, with a fructose/palmitic acid ratio 1, at atmospheric pressure in order to produce fructose monopalmitate. The progress of the reaction was monitored by TLC analysis.

**Fig. 6:**
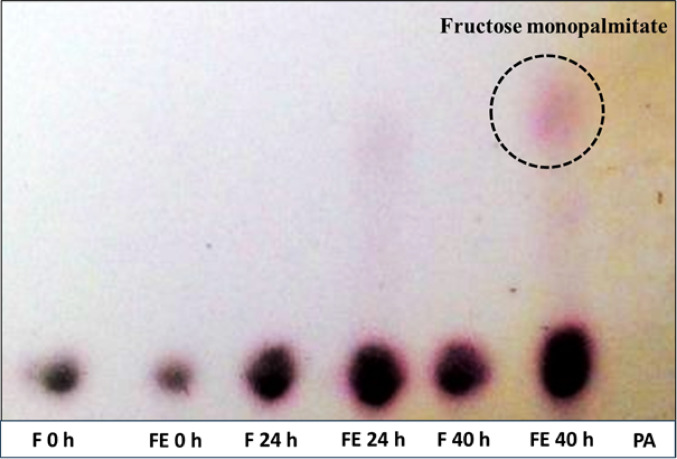
TLC of the fructose monopalmitate yield at 40th hour. F: Fructose; FE: Fructose with lipase; PA: Palmitic acid

TLC of lipase-catalyzed synthesis of fructose monopalmitate is shown in Fig. 6. After 40 h of reaction, fructose monopalmitate concentration slightly increased to 8.4% (w/w of the reaction mixture). The conversion percentage of fructose with palmitic acid leading to fructose monopalmitate is shown in Table 4.

Table 4 shows a steady but slow reaction rate, with conversion nearly doubling between 24 and 40 h. This indicates a gradual increase in product yield over time. Palmitic acid concentration decreases over time from 153.85 mg at the start of the reaction to 146.88 mg at 24 h and 140.99 mg at 40 h. Correspondingly, the moles of palmitic acid decrease from 0.60 mmol to 0.54 mmol, consistent with its consumption in the formation of the ester (fructose monopalmitate).

The conversion of fructose and palmitic acid into fructose monopalmitate appears to be moderate, as the yield remains relatively low (just 8.4%) even after a prolonged reaction time. Since the increase in concentration is described as slight, it may indicate that the reaction is approaching equilibrium, where further significant increases in product concentration are unlikely without changes to the reaction conditions.

The formation of fructose monopalmitate specifically (rather than other by-products) suggests some level of selectivity, but the low yield could be a limitation for industrial-scale production. The reaction likely proceeds slowly or may be limited by factors such as enzyme activity, mass transfer limitations, or substrate solubility. Overall, Table 4 demonstrates a modest but consistent progression in fructose monopalmitate formation over 40 h. However, the relatively low conversion percentage (8.4%) suggests a need for optimization of reaction conditions to improve yield and efficiency.

To increase the efficiency of fructose monopalmitate synthesis, various optimization strategies can be developed, considering both reaction conditions and catalysts. Temperature and reaction time can be optimized. Given that only 8.4% conversion was achieved in 40 h, longer reaction times may be tested to determine whether the conversion plateaus or continues to increase. Increasing the enzyme concentration or using immobilized enzymes could enhance the conversion rate but must be balanced against process costs.

Optimization of reaction conditions will be crucial for understanding the interactions and identifying the optimal parameters, which will be the focus of future work.

**Table 4:** Reaction performance of Fructose monopalmitate

Time, h	Area	Palmitic acid, mg	Palmitic acid, mmol	Conversion percentage, %
0	1,317,305,706	153.85	0.6	–
24	1,257,644,289	146.88	0.57	4.5
40	1,207,139,595	140.99	0.54	8.4

Non-enzymatic synthesis of the SFAEs have also been produced via chemical methods. However, these chemical approaches tend to be less efficient due to more energy consumption and exhibiting lower selectivity compared to enzymatic processes. Moreover, chemical synthesis often involves high temperatures and use of alkalines, which can lead to undesirable discoloration in the product and the formation of harmful byproducts. Some of these byproducts have been found to be allergenic or even potentially carcinogenic. Consequently, enzymatic synthesis using lipase is generally favored for producing sugar fatty acid esters due to its milder conditions and cleaner production process (Gumel et al. 2011).

## Conclusion

The biochemical characterization of the *Cryptococcus albidus* D24 lipase reveals its potential for industrial applications, particularly in the synthesis of sugar fatty acid esters. The stability of the enzyme in organic solvents and its positive response to certain metal ions make it a versatile catalyst in various industrial processes. The successful esterification reactions highlight the potential of the lipase for not only environmentally friendliness but also convenient industrial use, providing an alternative to traditional chemical methods. This work underscores the suitability of D24 lipase for future biotechnological processes, offering an efficient, stable, and sustainable enzyme solution for esterification reactions.

## Data Availability

No datasets were generated or analysed during the current study.

## References

[CR1] Abdelaziz AA, Abo-Kamar AM, Elkotb ES, Al-Madboly LA (2025) Microbial lipases: advances in production, purification, biochemical characterization, and multifaceted applications in industry and medicine. Microb Cell Fact 24(1):40. 10.1186/s12934-025-02664-6 39939876 10.1186/s12934-025-02664-6 PMC11823137

[CR2] Bharathi D, Rajalakshmi G (2019) Microbial lipases: an overview of screening, production and purification. Biocatal Agric Biotechnol 22:101368. 10.1016/j.bcab.2019.101368

[CR3] Bradford MM (1976) A rapid and sensitive method for the quantitation of microgram quantities of protein utilizing the principle of protein-dye binding. Anal Biochem 72(1–2):248–254 942051 10.1016/0003-2697(76)90527-3

[CR4] Cao L, Fischer A, Bornscheuer UT, Schmid RD (1996) Lipase-catalyzed solid phase synthesis of sugar fatty acid esters. Biocatal Biotransfor 14(4):269–283. 10.3109/10242429609110280

[CR5] de Almeida AF, Dias KB, da Silva AC, Terrasan CR, Tauk-Tornisielo SM, Carmona EC (2016) Agroindustrial wastes as alternative for lipase production by Candida viswanathii under Solid-State cultivation: purification, biochemical properties, and its potential for poultry fat hydrolysis. Enzyme Res 1353497. 10.1155/2016/1353497 10.1155/2016/1353497 PMC5048095 27725884

[CR6] Dosanjh NS, Kaur J (2002) Immobilization, stability and esterification studies of a lipase from a *Bacillus* Sp. Biotechnol Appl Biochem 36(1):7–12 12149117 10.1042/ba20010070

[CR7] Gumel AM, Annuar MSM, Heidelberg T, Chisti Y (2011) Lipase mediated synthesis of sugar fatty acid esters. Process Biochem 46(11):2079–2090. 10.1016/j.procbio.2011.07.021

[CR8] Gupta R, Kumari A, Syal P, Singh Y (2015) Molecular and functional diversity of yeast and fungal lipases: their role in biotechnology and cellular physiology. Prog Lipid Res 57:40–54. 10.1016/j.plipres.2014.12.001 25573113 10.1016/j.plipres.2014.12.001

[CR9] Kiran S, Arshad Z, Nosheen S, Kamal S, Gulzar T, Majeed MS, Jannat M, Rafique MA (2016) Microbial lipases: production and applications: a review. J Biochem Biotechnol Biomater 1(2):7–20. https://d1wqtxts1xzle7.cloudfront.net/68314365/2016_Kiran_et_al._2016-libre.pdf?1627296409=&response-content-disposition=inline%3B+filename%3DMicrobial_Lipases_Production_and_Applica.pdf&Expires=1749452304&Signature=fg699xn3XpnYMFpJNAUvTsB9OMeNmeiB-AAQrsYGCC~jvWx3dNOE9-auyBeYzGYd6h-H3wmluhekvpH3EXalViBZTD3~ti6dhg2xCezj5H65QoAqDQ2-SBCHl0uodLmUN-bzeajEuyEkRgAEz5OK~CiY3qtd8gk-Z0q5pkuUcp4x3XOG9v4ygRhkEidtFY5jm~UrdkaGJCDvTCX~hqZnu8PcFH8e7QoIiqsKw8L5gIO~pxXYkMaY~LDUxeqbGDOfzkR0sDSwsdXwzHEG-WtZrBEKZy33GncYsKjpViNhsT8SmfGRZFA1dDx8Y9kRYk2f42Vcae5UmCkxYTwu6EEO4w__&Key-Pair-Id=APKAJLOHF5GGSLRBV4ZA

[CR11] Kumar N (2012) Synthesis of Sugar Fatty Acid Esters using Lipase Immobilized in Supported Sol-Gels. Dissertation, University of Waterloo

[CR10] Kumar A, Verma V, Dubey VK, Srivastava A, Garg SK, Singh VP, Arora PK (2023) Industrial applications of fungal lipases: a review. Front Microbiol 14:1142536. 10.3389/fmicb.2023.1142536 37187537 10.3389/fmicb.2023.1142536 PMC10175645

[CR12] Lan D, Hou S, Yang N, Whiteley C, Yang B, Wang Y (2011) Optimal production and biochemical properties of a lipase from *Candida albicans*. Int J M Sci 12(10):7216–7237. 10.3390/ijms12107216 10.3390/ijms12107216 PMC3211034 22072943

[CR13] Li Z, Liu J, Fang Y, Chen H, Yang B, Wang Y (2024) An efficient and high-water-content enzymatic esterification method for the synthesis of β-sitosterol conjugated linoleate via a sodium citrate-based three-liquid-phase system. Food Chem 458:140250. 10.1016/j.foodchem.2024.140250 38964114 10.1016/j.foodchem.2024.140250

[CR14] Liu Z, Chi Z, Wang L, Li J (2008) Production, purification and characterization of an extracellular lipase from *Aureobasidium pullulans* HN2.3 with potential application for the hydrolysis of edible oils. Biochem Eng J 40(3):445–451. 10.1016/j.bej.2008.01.014

[CR15] Lohith K (2006) Enzymatic synthesis of selected amino acid esters of sugars. Dissertation, University of Mysore

[CR16] Lotti M, Pleiss J, Valero F, Ferrer P (2015) Effects of methanol on lipases: molecular, kinetic and process issues in the production of biodiesel. Biotechnol J 10(1):22–30. 10.1002/biot.201400158 25046365 10.1002/biot.201400158

[CR17] Lu J, Brigham CJ, Rha C, Sinskey AJ (2013) Characterization of an extracellular lipase and its chaperone from Ralstonia eutropha H16. Appl Microbiol Biotechnol 97:2443–2454. 10.1007/s00253-012-4115-z 22588499 10.1007/s00253-012-4115-z

[CR18] Mahfoudhi A, Benmabrouk S, Fendri A, Sayari A (2022) Fungal lipases as biocatalysts: A promising platform in several industrial biotechnology applications. Biotechnol Bioeng 119(12):3370–3392. 10.1002/bit.28245 36137755 10.1002/bit.28245

[CR19] Melani NB, Tambourgi EB, Silveira E (2020) Lipases: from production to applications. Sep Purif Rev 49(2):143–158. 10.1080/15422119.2018.1564328

[CR20] Najjar A, Hassan EA, Zabermawi N, Saber SH, Bajrai LH, Almuhayawi MS, Abujamel TS, Almasaudi SB, Azhar LE, Moulay M, Harakeh S (2021) Optimizing the catalytic activities of methanol and thermotolerant Kocuria flava lipases for biodiesel production from cooking oil wastes. Sci Rep 11(1):13659. 10.1038/s41598-021-93023-z 34211018 10.1038/s41598-021-93023-z PMC8249636

[CR42] Ogino H, Ishikawa H (2001). Enzymes which are stable in the presence of organic solvents. Journal of bioscience and bioengineering, 91(2), 109-116. https://www.sciencedirect.com/science/article/abs/pii/S1389172301800517 10.1263/jbb.91.109 16232960

[CR21] Pérez B, Anankanbil S, Guo Z (2017) Synthesis of sugar fatty acid esters and their industrial utilizations. Fatty acids, pp 329–354

[CR22] Rapp P, Backhaus S (1992) Formation of extracellular lipases by filamentous fungi, yeasts, and Bacteria. Enzyme Microb Technol 14(11):938–943. 10.1016/0141-0229(92)90059-W

[CR23] Romo-Silva M, Flores-Camargo EO, Chávez-Camarillo GM, Cristiani-Urbina E (2024) Production, purification, and characterization of extracellular lipases from Hyphopichia wangnamkhiaoensis and Yarrowia deformans. Fermentation 10(12):595. 10.3390/fermentation10120595

[CR24] Šabeder S, Habulin M, Knez Ž (2006) Lipase-catalyzed synthesis of fatty acid Fructose esters. J Food Eng 77(4):880–886. 10.1016/j.jfoodeng.2005.08.016

[CR25] Santambrogio C, Sasso F, Natalello A, Brocca S, Grandori R, Doglia SM, Lotti M (2013) Effects of methanol on a methanol-tolerant bacterial lipase. Appl Microbiol Biotechnol 97:8609–8618. 10.1007/s00253-013-4712-5 23371296 10.1007/s00253-013-4712-5

[CR28] Sharma S, Kanwar SS (2014) Organic solvent tolerant lipases and applications. Sci World J, 2014(1), 625258. 10.1155/2014/625258 10.1155/2014/625258 PMC3929378 24672342

[CR27] Sharma R, Chisti Y, Banerjee UC (2001) Production, purification, characterization, and applications of lipases. Biotechnol Adv 19(8):627–662. 10.1016/S0734-9750(01)00086-6 14550014 10.1016/s0734-9750(01)00086-6

[CR26] Sharma AK, Sharma V, Saxena J (2016) A review paper on properties of fungal lipases. Int J Curr Microbiol App Sci 5(12):123–130. 10.20546/ijcmas.2016.512.014

[CR29] Sifour M, Saeed HM, Zaghloul TI, Berekaa MM, Abdel-Fattah YR (2010) Purification and properties of a lipase from thermophilic *Geobacillus stearothermophilus* strain-5. Int J Biol Chem 4(4):203–212 10.1016/j.nbt.2010.04.004 20412872

[CR30] Singh B, Jana AK (2023) Agri-residues and agro-industrial waste substrates bioconversion by fungal cultures to biocatalyst lipase for green chemistry: A review. J Environ Manage 348:119219. 10.1016/j.jenvman.2023.119219 37852078 10.1016/j.jenvman.2023.119219

[CR31] Somashekar BR, Divakar S (2007) Lipase catalyzed synthesis of L-alanyl esters of carbohydrates. Enzyme Microb Technol 40(2):299–309

[CR41] Talley K, Alexov E (2010). On the pH‐optimum of activity and stability of proteins. Proteins: Structure, Function, and Bioinformatics, 78(12), 2699-2706. 10.1002/prot.22786 10.1002/prot.22786 PMC2911520 20589630

[CR33] Tiwari P, Upadhyay MK, Silawat N, Verma HN (2011) Optimization and characterization of a thermo tolerant lipase from *Cryptococcus albidus*. Der Pharma Chem 3(4):501–508

[CR34] Tracy P, Dasgupta D, More S (2023) Challenges and opportunities for production of C5 sugar fatty acid esters (SFAEs) from renewable resources. Ind Crops Prod 193:116170

[CR35] Vakhlu J, Kour A (2006) Yeast lipases: enzyme purification, biochemical properties and gene cloning. Electron J Biotechnol 9(1). 10.2225/vol9-issue1-fulltext-9

[CR36] Yalçin HT, Çorbaci C, Uçar FB (2014) Molecular characterization and lipase profiling of the yeasts isolated from environments contaminated with petroleum. J Basic Microbiol 54:85–92. 10.1002/jobm.201300029 10.1002/jobm.201300029 23712936

[CR39] Yilmaz DE (2016) Biochemical characterization of newly identified lipases for utilization in lipase catalysed reactions, Dissertation, Marmara University

[CR37] Yilmaz DE, Sayar NA (2015) Organic solvent stable lipase from *Cryptococcus diffluens* D44 isolated from petroleum sludge. J Mol Catal B Enzym 122:72–79. 10.1016/j.molcatb.2015.08.021

[CR38] Yilmaz DE, Yalçın HT, Sayar NA (2014) Screening of lipase production among different microorganisms. N Biotechnol 31S90. 10.1016/j.nbt.2014.05.1821

[CR40] Zheng Y, Zheng M, Ma Z, Xin B, Guo R, Xu X (2015) Sugar fatty acid esters. Polar lipids. Elsevier, pp 215–243

